# Australians’ willingness to change their discretionary food intake: findings from the CSIRO junk food analyser

**DOI:** 10.3389/fpubh.2024.1385173

**Published:** 2024-05-15

**Authors:** Chelsea E. Mauch, Emily Brindal, Gilly A. Hendrie

**Affiliations:** Human Health Program, Health and Biosecurity, CSIRO, Adelaide, SA, Australia

**Keywords:** discretionary food, behaviour change, stage of change, willingness, dietary intake

## Abstract

**Introduction:**

Overconsumption of energy dense, nutrient poor foods and beverages is a major problem globally. This study describes what and how Australian adults consume and are willing to change their intake in terms of discretionary food and beverage categories.

**Methods:**

Cross-sectional data were collected via the CSIRO Junk Food Analyser. This online tool contains short questions on discretionary food and beverage consumption, and items relating to stage of and willingness and strategies to reduce discretionary food and beverage intake. Analyses focussed on describing discretionary intake, adherence to guidelines and the prediction of willingness to change discretionary food intake amongst those exceeding guidelines.

**Results:**

In 2021, 41,109 Australian adults completed the CSIRO Junk Food Analyser. Participants were mostly female (73.1%) and aged 31–70 years (78.9%). Most participants exceeded dietary guidelines for discretionary food and beverage intake (67.4%, 27,694/41,109) with 40% reporting actively trying to reduce intake. Most people exceeding guidelines did so in categories of alcohol (39.3%) and cakes and biscuits (21.0%). Yet, willingness to change intake was lowest for alcohol (median, IQR of 3, 2:4 out of 5). Almost half of the participants were willing to try ‘having a few days off per week’ (46.0%), while only 13.4% were willing to try to ‘eliminate’ their highest ranked category.

**Discussion:**

Australian adults are willing to reduce their discretionary food and beverage intake, but simply targeting the foods and beverages consumed most may not be the best place to start. Messages encouraging days off frequently consumed discretionary foods and beverages may be well received.

## 1 Introduction

The consumption of energy dense, nutrient poor foods and beverages is a major problem globally. ‘Discretionary’ foods and beverages, as they are defined in Australia, are foods and beverages that are high in energy, saturated fat, added sugars, salt and/or alcohol, and are unnecessary for good health (1). Overconsumption of discretionary food and beverages is common in Australia, with intakes generally exceeding 30% of total energy intake across the population (2). Globally, reported intakes are similar, with data from North America and the United Kingdom showing similarly excessive intakes of energy dense, nutrient poor foods (3–5). With their high energy density, excessive intake of discretionary foods and beverages contributes to the incidence of obesity and non-communicable diseases such as cardiovascular disease, diabetes and cancer (6, 7). The cost of diet-related disease in terms of morbidity, mortality and quality of life lost, along with the economic impacts related to poor health and loss of productivity are vast (7). This public health nutrition challenge is therefore of high priority to the Australian Government, with the National Preventive Health Strategy setting the ambitious target to reduce the proportion of energy from discretionary foods and beverages to less than 20% by 2030 (8).

The discretionary food and beverage category includes a diverse range of items with varying sensory properties, but with sweet, salt, and/or fat predominating. Our innate preference for these tastes (9), along with the ingrained social conventions (10) and pleasure attached to consuming these foods and beverages, makes changing intake of discretionary food and beverages particularly complex and challenging. Perhaps for that reason, discretionary food and beverages are not commonly targeted in public health campaigns and interventions, which tend to have a stronger focus on increasing intakes of healthy foods such as fruit and vegetables (11, 12). Regardless, there has been some improvement at a population level over the last two decades, with some reduction in total daily serves or portions of discretionary foods and beverages across children, adolescents and adults aged up to 50 years (13). This has been largely due to reductions in the consumption of sugar-sweetened beverages (SSB’s) and other sweet discretionary foods, likely driven by stronger public messaging resulting in an increased awareness of the impacts of sugar on health (14). This indicates that population change in this space is possible, but there remains more to be done to achieve the targets set out by the National Preventive Health Strategy. Understanding the public’s perspective on discretionary food and beverages, including where they currently stand with changing their intake, what they are willing to change and strategies they are willing to try in order to reduce their intake is important in forming effective programmes and campaigns in the future.

The public is generally aware of the need to limit their intake of discretionary foods and beverages in order to achieve good health, and have shown some interest in understanding how their intake compares to dietary guideline recommendations. In April 2021, the Commonwealth Scientific and Industrial Research Organisation (CSIRO) launched the Junk Food Analyser (JFA) (15). The JFA is a freely available, online tool designed to support the Australian public to understand their intake of discretionary food and beverages and how it compares with the Australian Dietary Guidelines (ADG’s) age and sex specific maximum daily servings recommendations (1). There was strong media and public interest in the tool, with over 40 thousand Australian adults using it within the 10 weeks after its launch. The tool collected important information about both the intake of discretionary foods and beverages, and the public’s perspectives on changing their intake. The aim of this paper is to describe what and how Australian adults are willing to change with respect to their intake of discretionary food and beverages. More specifically, it will examine adults readiness to change their intake, examine which categories of discretionary food and beverages Australian adults are most willing to change, and explore the strategies adults are willing to try in order to reduce their intake of discretionary food and beverages.

## 2 Materials and methods

### 2.1 Study design and setting

This paper uses data collected through the JFA online tool, which included a series of survey questions. The JFA was developed in collaboration with Digital Wellness (Sydney, Australia), a business dedicated to delivering digital health platforms. The JFA was launched via media release on the 7th of April 2021, resulting in numerous television, radio, print, online and social media articles and mentions, reaching an estimated 10.7 million people. Data for the current study was collected within the first 10 weeks after launch. This study was approved by the CSIRO Health and Medical Human Research Ethics Committee Low Risk Review Panel (2021_035_LR).

#### 2.1.1 Participants

Participants included Australian adults aged at least 18 but no more than 100 years, and with a body mass index (BMI) of at least 18.5 kg/m^2^ (i.e., not classified as underweight). People with a BMI below 18.5 kg/m^2^ are classified as underweight and would generally not be advised to reduce their intake of any food group. They were therefore excluded from analysis. Participants with extreme values for weight (less than 13 and greater than 250 kg), height (less than 1 and greater than 3 metres), BMI (greater than 97 kg/m^2^) or discretionary food and beverage intake (see details below), those with missing discretionary food and beverage data and duplicate records were excluded.

#### 2.1.2 Survey items

Discretionary food and beverage survey items from the CSIRO Healthy Diet Score survey were used to assess intake of 11 different categories of foods and beverages (16). Details of the development and validation of the Healthy Diet Score questions have been published elsewhere (16, 17). Categories are consistent examples of discretionary foods and beverages provided in the Australian Dietary Guidelines and include cakes and biscuits, confectionary, takeaway foods (including commercial pizza, burgers and fried chicken), SSB’s, alcoholic beverages (including beer, wine and spirits), fried potato (including fried hot chips or fries), savoury snacks (including potato crisps), savoury pastries (such as pies and pasties), muesli and cereal bars, ice-cream and ice lollies, and processed meat (such as sausages and salami). Each category has a frequency and portion-based question used to estimate daily intake in serves (termed ‘portions’ in the United Kingdom). Demographic survey items included sex (male or female), year of birth, highest level of education (primary school, year 12 or equivalent, TAFE or technical college certificate, bachelor’s or post-graduate degree), and self-reported weight and height. Items regarding the modification of discretionary food and beverage intake included stage of change, willingness and strategies. The stage of change survey item was based on broader theoretical models of processes and change. It asked participants ‘Where do you currently stand with consuming fewer…..’ in relation to their highest ranked category of discretionary food or beverage and included nine response options; ‘feel like I am successfully doing it’, ‘am trying and making progress’, ‘have tried and am still trying’, ‘recently started doing something’, ‘have decided to do something, but not started’, ‘have tried and am planning to try again’, ‘have tried and will not try again’, ‘decided there was no need to do anything’ and ‘have not given it much thought / undecided’. Willingness to change intake of discretionary food and beverages was assessed with a single item asking participants to rate how willing they would be to change their intake of each discretionary food and beverage category they consume on a 5-point Likert scale from ‘not at all willing to try’ to ‘very willing to try’. Participants were asked which strategies they would be willing to try to reduce their intake, but only for the category ranked highest in terms of intake. Response options included:

1. Eliminating the discretionary food or beverage category from their diet entirely.
2. Halving the amount consumed on each occasion.
3. Having a few days per week where they do not consume the category.
4. Having fewer types of the discretionary food or beverage category.
5. Swapping foods or beverages for healthier items.

Participants could also respond with ‘None, I do not want to try to reduce my intake of these items’ (*n* = 1,450), or with ‘My own strategy which is not listed here’ (*n* = 534), however these results are not presented here.

#### 2.1.3 Data preparation

Daily serves of each discretionary food and beverage category was calculated and adjusted in order to address self-report bias (18). Total daily intake of discretionary food and beverages was then calculated by summing daily adjusted serves of all 11 categories. An individuals’ highest ranked category was considered the category of discretionary food or beverage with the highest adjusted daily servings. This was automatically calculated during survey completion and used to personalise the questions regarding stage of change and strategies. However, during the data checking phase it was noted that in *n* = 528 cases, the highest ranked category was incorrectly allocated for the purpose of personalising these questions, therefore these participants were excluded in analyses using stage of change or strategies questions.

Extreme over-reporters of discretionary food and beverage intake were identified by applying a plausible reporters cut-off (19). Participants reporting to consume an energy intake from discretionary food and beverages that was greater than 2.75 times their basal metabolic rate (based on those used in the Nutrient Reference Values) (20) were then excluded.

BMI was calculated using weight in kilograms and height in metres, and converted to weight status using World Health Organization International Classifications for adults (21). Year of birth was used to determine age in years and grouped similarly to the Nutrient Reference Value age groupings (18–30, 31–50, 51–70, 71+ years) (1). Highest level of education was collapsed into a dichotomous variable of *university* (‘bachelor’s or post-graduate degree’) and *no university* (‘primary school’, ‘year 12 or equivalent’, and ‘TAFE or technical college certificate’). The stage of change items were collapsed into five stages of change; *success* (‘feel like I am successfully doing it’), *active* (‘am trying and making progress’ and ‘have tried and am still trying’), *early stages* (‘recently started doing something’ and ‘have decided to do something, but not started’), *planning to start again* (‘have tried and am planning to try again’) and *disengaged / have not thought about it* (‘have tried and will not try again’, ‘decided there was no need to do anything’ and ‘have not given it much thought / undecided’).

#### 2.1.4 Data analysis

Demographic characteristics of the sample were assessed using count and percent, or median and interquartile range (IQR), and presented for the total sample, along with the samples exceeding or consuming within the ADG’s age and sex specific maximum recommended daily serves (1). The remainder of the analyses focused on the sample exceeding these guidelines. Stage of change and strategies for reducing intake were presented by highest ranked category, with the proportion of participants at each stage of change or indicating a willingness to try the strategy determined. Median (IQR) willingness to change across all consumers of each category of discretionary food and beverages was determined, along with the proportion of consumers willing or very willing (i.e., a 4 or 5 on the 5-point Likert scale) to reduce intake of their highest ranked category. Multiple regression was used to explore the determinants of mean willingness to change, with sex (male as the reference category), age (in years), education (no university degree as the reference category), BMI (in kg/m^2^), total daily discretionary intake (in serves) and highest ranked category (dummy coded, with alcohol as the reference category) as predictor variables. Significance was set at the *p* < 0.05 level, while standardised beta (β) values were used to compare the relative importance of predictors, and adjusted R^2^ the amount of variance explained by the model. Willingness to change intake of the highest ranked category was also compared to mean willingness across the remaining categories using paired samples *t*-tests.

## 3 Results

### 3.1 Sample description

The JFA was completed 42,327 times in the 10 weeks following launch. Of these, 1,218 were duplicate records, had implausible BMI, weight, height, age or energy intake, were classified as underweight, or had missing data on one or more discretionary food and beverage categories. A total of 41,109 participants were included, being mostly female (30,041/41,109, 73.1%), aged between 31 to 70 years (32,421/41,109, 78.9%), and university educated (23,863/38,788, 61.5%; Table 1). Almost two thirds of participants were classified as having overweight or obesity (13,791/41,109, 33.5% and 12,126/41,109, 29.5% respectively). Alcohol, followed by cakes and biscuits, and confectionary were the categories consumed in the greatest quantities, with 37.1% (15,224/41,004) of the sample having alcohol as their highest ranked intake category according to serves, and 20.0 and 12.9% having cakes & biscuits or confectionary as their highest ranked category, respectively. Participants consumed a median (IQR) of 8(7, 10) different categories of discretionary food and beverages, and 3.65 (2.08, 5.93) total serves per day. Two thirds of participants (67.4%, 27,694/41,109) exceeded the maximum recommended amount of discretionary food and beverages in the ADG’s. Those exceeding the guidelines included a greater proportion of men (28.8% vs. 23.0%), younger and middle-aged adults (14.7% vs. 13.1% 18–30 year-olds, and 38.3% vs. 34.0% 31–50 year-olds), people without a university education (41.0% vs. 33.3%), and people classified as having obesity (32.7% vs. 22.8%) than those not exceeding the guidelines. They also consumed around two more categories than those not exceeding guidelines, but their overall willingness to change intake (median across all discretionary food and beverage categories) was similar. The remainder of the results will focus on the sample of participants exceeding the maximum recommended amount of discretionary food and beverage guidelines (*n* = 27,694) as these people would benefit most from a reduction in intake.

**Table 1:** Description of the sample completing the junk food analyser tool (*N* = 41,109)^a^.

Variable	Category	Total sample (*N* = 41,109)^b^	Exceeding guidelines^c^ (*n* = 27,694)^d^	Not exceeding guidelines^c^ (*n* = 13,415)^e^
		*n*(%)	*n*(%)	*n*(%)
Sex	Female	30,041 (73.1)	19,715 (71.2)	10,326 (77.0)
	Male	11,068 (26.9)	7,979 (28.8)	3,089 (23.0)
Age groups	18–30 years	5,829 (14.2)	4,069 (14.7)	1,760 (13.1)
	31–50 years	15,155 (36.9)	10,598 (38.3)	4,557 (34.0)
	51–70 years	17,266 (42.0)	11,127 (40.2)	6,139 (45.8)
	71+ years	2,859 (7.0)	1,900 (6.9)	959 (7.1)
Highest level of education	No university	14,925 (38.5)	10,707 (41.0)	4,218 (33.3)
	University	23,863 (61.5)	15,412 (59.0)	8,451 (66.7)
Weight status	Healthy weight	15,192 (37.0)	9,175 (33.1)	6,017 (44.9)
	Overweight	13,791 (33.5)	9,450 (34.1)	4,341 (32.4)
	Obese	12,126 (29.5)	9,069 (32.7)	3,057 (22.8)
Highest ranked category of intake	Alcohol	15,224 (37.1)	10,887 (39.3)	4,337 (32.6)
	Cakes & biscuits	8,195 (20.0)	5,822 (21.0)	2,373 (17.8)
	Confectionary	5,270 (12.9)	3,464 (12.5)	1,806 (13.6)
	Takeaway	4,275 (10.4)	2,465 (8.9)	1,810 (13.6)
	Processed meat	2,276 (5.6)	1,413 (5.1)	863 (6.5)
	SSBs	1,485 (3.6)	1,220 (4.4)	265 (2.0)
	Ice-cream	1,320 (3.2)	751 (2.7)	569 (4.3)
	Savoury snacks	1,085 (2.6)	672 (2.4)	413 (3.1)
	Savoury pastries	803 (2.0)	521 (1.9)	282 (2.1)
	Bars	669 (1.6)	305 (1.1)	364 (2.7)
	Fried potato	402 (1.0)	174 (0.6)	228 (1.7)
		Median (IQR)	Median (IQR)	Median (IQR)
No categories consumed (of max 11)		8.00 (7.00:10.00)	9.00 (8.00:10.00)	7.00 (5.00:8.00)
TOTAL discretionary intake—serves		3.65 (2.08:5.93)	4.95 (3.61:7.30)	1.60 (0.98:2.05)
Highest ranked category—serves (consumers)		1.59 (0.84:2.65)	2.07 (1.39:3.22)	0.60 (0.43:0.91)
Overall willingness to change discretionary intake^f^		4.00 (3.40:4.50)	4.00 (3.43:4.44)	4.00 (3.38:4.63)

^a^All samples exclude extreme BMI, weight, height, age and discretionary intake, those missing discretionary choice data, duplicate records, and those classified as underweight.^b^*n* = 2,321 missing due to non-response for highest level of education, and *n* = 105 excluded from intake related items due to non-consumption of discretionary food and beverages.^c^ADG’s maximum recommended number of daily serves of discretionary food and beverages(1).^d^*n* = 1,575 with missing data due to non-response for highest level of education.^e^*n* = 746 missing due to non-response for highest level of education, and *n* = 105 excluded from intake related items due to non-consumption of discretionary food and beverages.^f^Willingness score—between 1 and 5, with higher score indicating a higher level of willingness.

### 3.2 Stage of change

**Figure 1:**
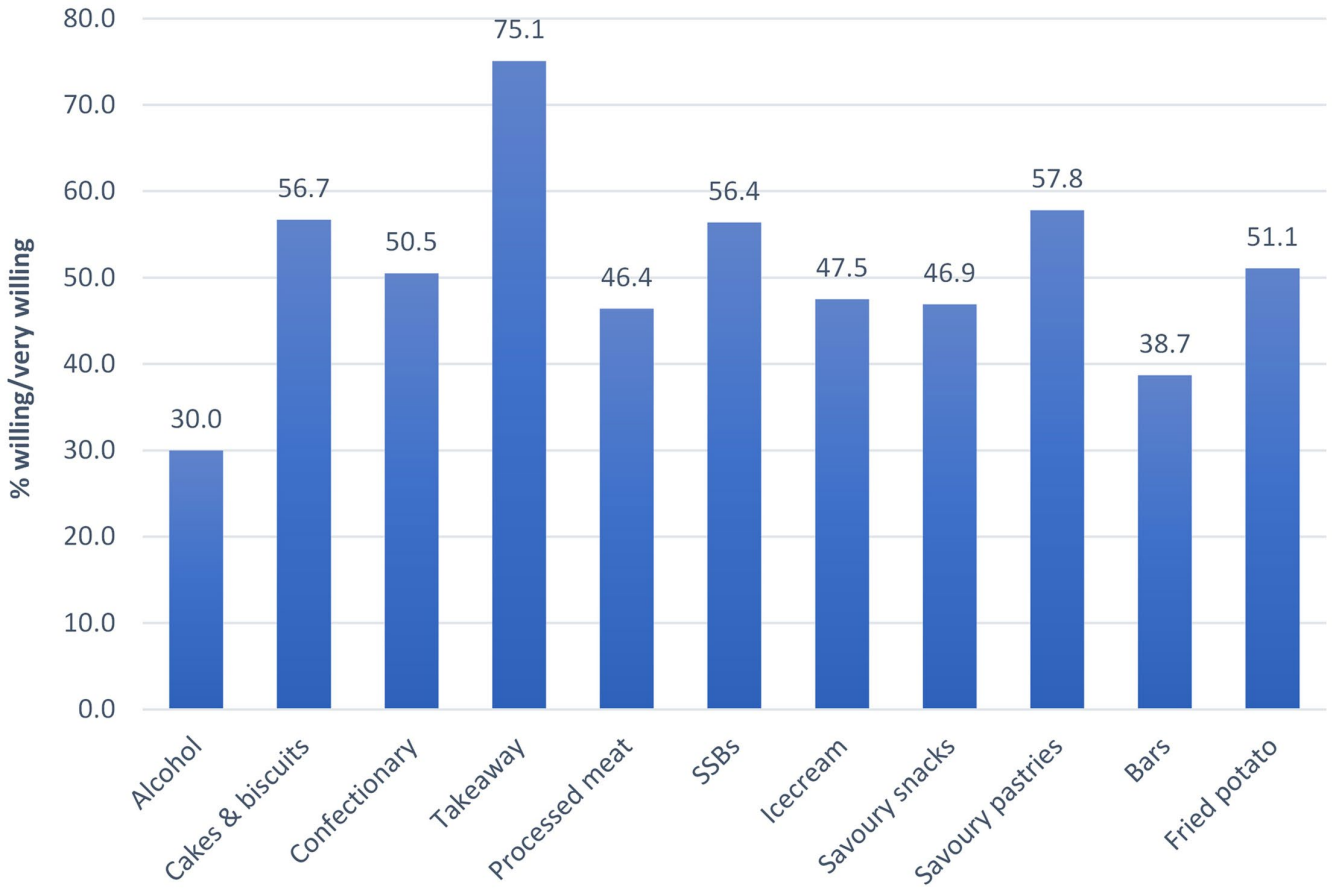
Proportion of participants exceeding guidelines that were willing or very willing to change intake of their highest ranked category (*n* = 27,693 excluding *n* = 1 with missing willingness data for their highest ranked category).

Only 6.6% (1,780/27,166) of participants reported that they were currently feeling successful in reducing intake of their highest ranked category, while 40.3% (10,956/27,166) were actively trying to reduce their intake (Figure 1; Supplementary Table 1). Close to 50% (1,178/2,465) of people with takeaway as their highest ranked category reported that they were actively trying to eat less takeaway, while around 30% of those consuming processed meat, ice-cream, savoury pastries, and bars as their highest ranked categories were actively trying to reduce their intake. Around 40% of those consuming processed meat (566/1,363) and bars (126/300) as their highest ranked foods were disengaged or had not considered eating less.

### 3.3 Willingness to change intake

**Figure 2:**
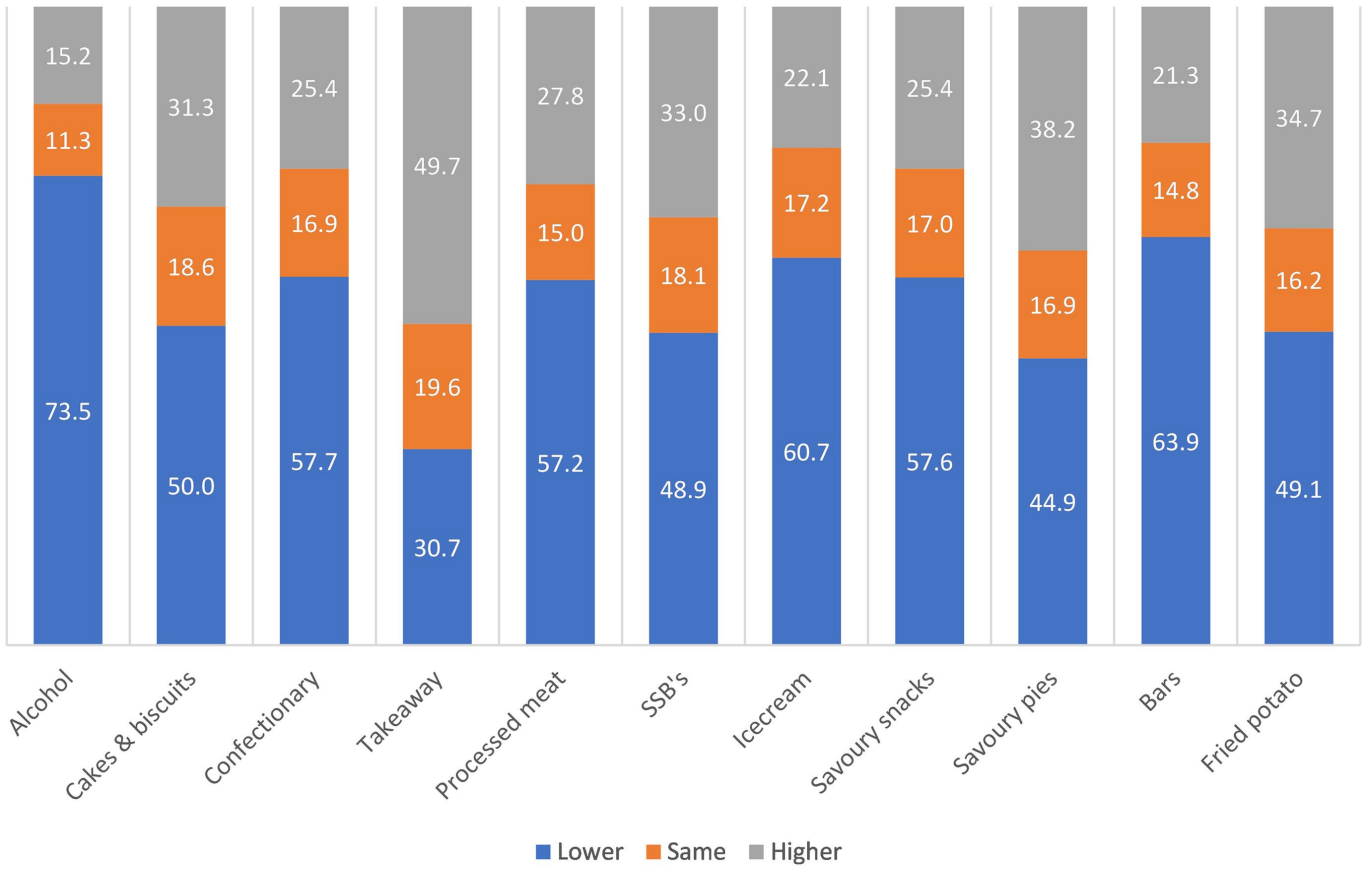
Proportion of participants exceeding guidelines that were willing or very willing to change intake of their highest ranked category (*n* = 27,693 excluding *n* = 1 with missing willingness data for their highest ranked category).

Overall willingness (across all consumers) to change intake was highest for takeaway foods and savoury pastries, with a median (IQR) score of 4 (4:5) out of a possible 5, and lowest for alcohol with a score of 3 (2:4; Table 2). Three quarters of those with takeaway as their highest ranked category were willing or very willing to change their intake (Figure 2). Just under 60% of those with cakes & biscuits, SSBs or savoury pastries as their highest ranked category were willing or very willing to change their intake. Whereas only 30% of those with alcohol as their highest ranked category were willing or very willing to change their intake.

**Table 2:** Willingness to change intake of discretionary food and beverage categories across all participants exceeding guidelines (*n* = 27,694).

Discretionary choice category	Median willingness (IQR)	*n* (%) consuming the category
Alcohol	3.0 (2.0:4.0)^a^	22,065 (79.7)
Cakes & biscuits	4.0 (3.0:5.0)	25,464 (91.9)
Confectionary	4.0 (3.0:5.0)	26,043 (94.0)
Takeaway	4.0 (4.0:5.0)	24,374 (88.0)
Processed meat	4.0 (3.0:5.0)	24,289 (87.7)
SSBs	4.0 (3.0:5.0)	13,452 (48.6)
Ice-cream	4.0 (3.0:5.0)	21,778 (78.6)
Savoury snacks	4.0 (3.0:5.0)	24,259 (87.6)
Savoury pastries	4.0 (4.0:5.0)	19,685 (71.1)
Bars	4.0 (3.0:5.0)	10,797 (39.0)
Fried potato	4.0 (3.0:5.0)	24,826 (89.6)

^a^Excluding *n* = 1 with missing willingness data for their highest ranked category, alcohol.

Mean willingness per participant (across all categories of discretionary food and beverages) was most strongly predicted by sex and BMI, with females and those with a higher BMI reporting higher overall willingness to change their intake (Table 3). Total daily intake of discretionary food and beverages was not associated with willingness. The model accounted for only 5.8% of variation in willingness to change intake.

**Table 3:** Multiple regression of mean willingness to change across all discretionary categories among participants exceeding guidelines (*n* = 26,119^a^).

Variable	B	SE	*β*
Sex (ref: male)	0.27	0.01	0.16***
Age (years)	0.00	0.00	0.03***
Highest level of education (ref: no uni)	−0.09	0.01	−0.06***
BMI (kg/m^2^)	0.02	0.00	0.15***
Discretionary intake (serves)	0.00	0.00	0.00
**Highest ranked category (ref: Alcohol)**			
Cakes & biscuits	0.01	0.01	0.01
Confectionary	0.06	0.02	0.03***
Takeaway	0.12	0.02	0.04***
Processed meat	−0.02	0.02	−0.01
SSBs	−0.03	0.02	−0.01
Ice-cream	−0.02	0.03	−0.00
Savoury snacks	0.02	0.03	0.00
Savoury pastries	0.01	0.04	0.00
Bars	0.04	0.05	0.01
Fried potato	−0.06	0.06	−0.01
Adjusted *R*^2^	0.058***		

*p < 0.05; **p < 0.01; ***p < 0.001.^a^Excludes *n* = 1,575 with missing data for highest level of education.

**Figure 3:**
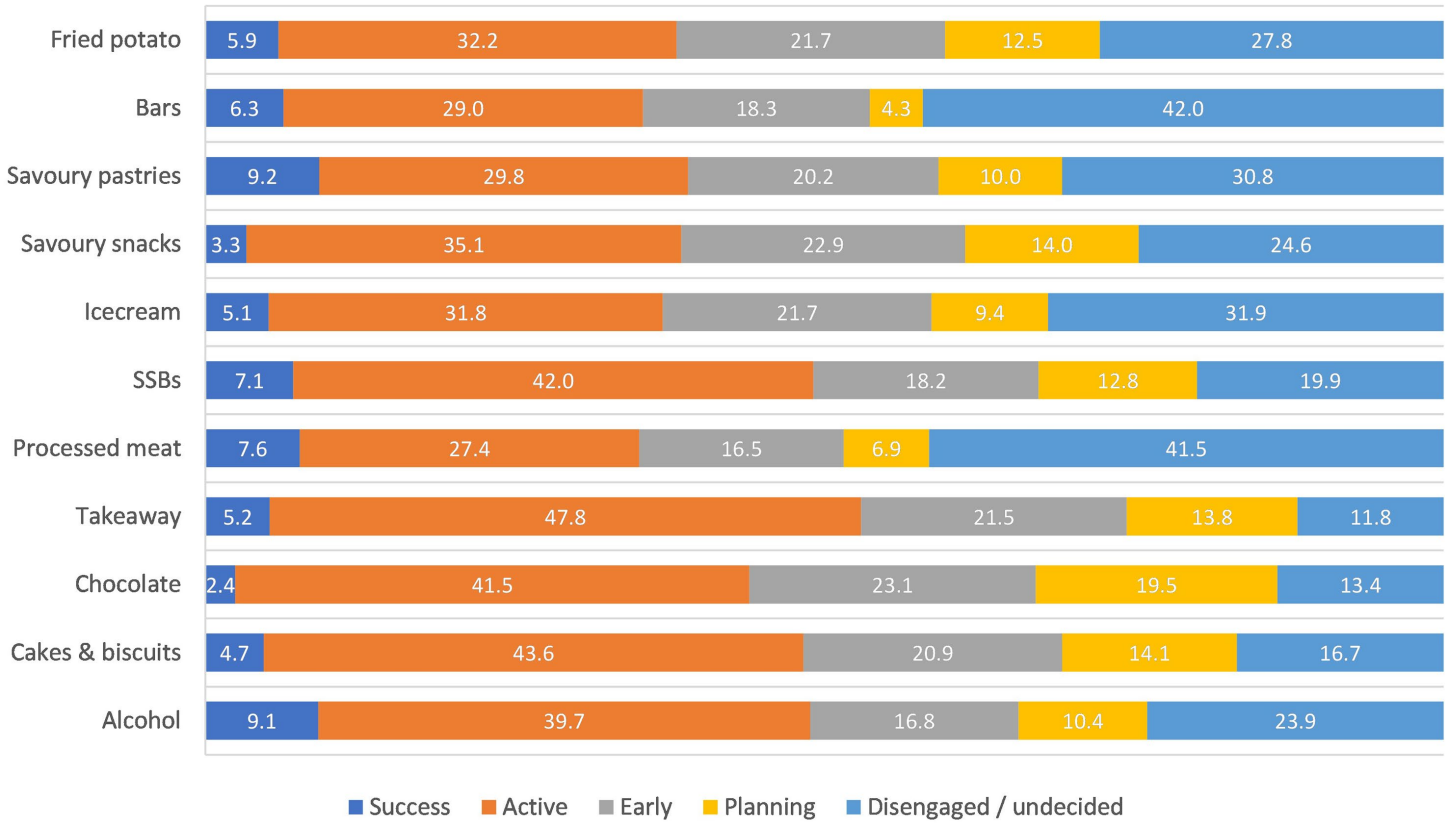
Proportion at each stage of change by highest ranked intake category amongst participants exceeding guidelines (*n* = 27,166 excluding *n* = 528 with incorrectly assigned question).

Willingness to change intake of the highest ranked category of discretionary food and beverages was generally lower or the same as mean willingness across all remaining categories (Figure 3; Supplementary Table 2), meaning that participants were slightly less willing to change their intake of the foods and beverages they consumed the most. Over 70% of those with alcohol as their highest ranked category had lower willingness to change their intake of alcohol than their mean willingness across all other categories. Whereas only 30% of those with takeaway as their highest ranked category had lower willingness to change their intake of takeaway than their mean willingness across all other categories. When comparing willingness to change intake of the highest ranked category to mean willingness across all remaining groups using paired samples *t*-tests, willingness for the highest ranked category was lower in all cases except where takeaway was the highest ranked category (Supplementary Table 3).

### 3.4 Strategies for reducing intake

Almost half of the participants (12,498/27,166, 46.0%) indicated that they would be willing to reduce their intake of their highest ranked category by ‘having a few days off per week’ (Table 4). This strategy was particularly preferred with respect to alcohol (58.6% of consumers with alcohol as their highest ranked category), confectionary (47.0%) and ice-cream (40.7%). Almost 50% of those consuming savoury snacks, takeaway or bars as their highest ranked category reported that they would be willing to swap those foods with a healthier alternative. Elimination was the least preferred strategy across all discretionary food or beverage categories, except for savoury pastries.

**Table 4:** Strategies that participants exceeding guidelines are willing to try to reduce highest ranked category of discretionary food and beverages (*n* = 27,166^a^).

Discretionary choice category	Strategies^b^					Sample with category ranked highest
	Eliminate	Halve	Days off	Fewer types	Healthy swaps	
Alcohol	855 (8.0)	2,964 (27.7)	6,262 (58.6)	2,058 (19.3)	1,762 (16.5)	10,689 (100.0)
Cakes & biscuits	874 (15.0)	1,781 (30.6)	2,265 (38.9)	1,587 (27.3)	2,381 (40.9)	5,816 (100.0)
Confectionary	650 (19.4)	1,188 (35.4)	1,578 (47.0)	897 (26.7)	1,273 (38.0)	3,354 (100.0)
Takeaway	487 (19.8)	835 (33.9)	880 (35.7)	916 (37.2)	1,149 (46.6)	2,465 (100.0)
Processed meat	181 (13.3)	347 (25.5)	448 (32.9)	350 (25.7)	505 (37.1)	1,363 (100.0)
SSBs	211 (17.9)	379 (32.2)	295 (25.1)	270 (23.0)	459 (39.0)	1,176 (100.0)
Ice-cream	112 (15.6)	165 (22.9)	293 (40.7)	113 (15.7)	222 (30.8)	720 (100.0)
Savoury snacks	101 (15.8)	191 (29.8)	243 (37.9)	151 (23.6)	300 (46.8)	641 (100.0)
Savoury pastries	103 (21.0)	130 (26.5)	91 (18.6)	141 (28.8)	168 (34.3)	490 (100.0)
Bars	48 (16.0)	54 (18.0)	95 (31.7)	53 (17.7)	136 (45.3)	300 (100.0)
Fried potato	24 (15.8)	54 (35.5)	48 (31.6)	39 (25.7)	49 (32.2)	152 (100.0)
Total sample	3,646 (13.4)	8,088 (29.8)	12,498 (46.0)	6,575 (24.2)	8,404 (30.9)	27,166 (100.0)

^a^Excludes *n* = 528 with incorrectly assigned question.^b^Multiple response question.

## 4 Discussion

This study aimed to determine what and how Australian adults are willing to change with respect to their discretionary food and beverage intake. In Australia, the dietary guidelines recommend limiting our consumption of discretionary foods and beverages which are those high in saturated fat, salt, sugar and alcohol, and are not an essential part of a healthy dietary pattern but can be included for variety and enjoyment. Overconsumption of discretionary foods is a common driver of poor diet quality globally, and consistently National dietary intake data shows an overconsumption of these energy-dense, nutrient-poor unhealthy foods and beverages (13, 16, 22, 23). Indeed, two thirds of participants surveyed in this study exceeded the maximum recommended amount of discretionary food and beverages in the Australian Dietary Guidelines, highlighting overconsumption of these foods as a key area for intervention to improve diet quality. Importantly, there was strong public engagement with the online Junk Food Analyser, demonstrating significant interest in discretionary food and beverage intake in the community. Findings provide insights into some of the ways that excessive intake of discretionary food and beverages can be addressed. We showed that people are less willing to change their intake of the discretionary foods and beverages they consume the most. Willingness to change intake was therefore lowest for one of the biggest problem categories, alcohol, while there was a higher willingness to address intake of takeaway foods. Generally, elimination was not a preferred strategy to reducing discretionary food and beverage intake. However, there was an interest in ‘days off’ as a strategy to reduce intake of more habitually consumed alcohol and sweet treats, whereas ‘healthy swaps’ was preferred for reducing intake of takeaway, savoury snacks and bars.

The Junk Food Analyser received a rapid and significant response following its launch via mainstream media release. This, combined with stage of change and willingness data, shows an overall interest from the Australian public in understanding this population wide dietary issue. Our survey showed that most over-consumers of discretionary food and beverages (around three quarters) were planning to, or in the early active stages of trying to reduce their intake. This proportion was highest for those with takeaway as their highest ranked intake category. Understanding people’s stages of change is useful for describing the population and providing tailored advice. However, its importance for predicting greater success with behaviour change is questionable (24). Willingness and behavioural intention are much stronger predictors of behaviour (25, 26), but literature suggest an intention-behaviour gap exists and needs addressing. For example, in physical activity, Rhodes and DeBrujn report a 48% intention-behaviour gap where people with intention fail to act to change their physical activity behaviour (27). In the current study in relation to dietary behaviour, participants were mostly willing and actively attempting to reduce their intake of discretionary foods and beverages, however literature would suggest there remains a challenge in bridging the willingness to action gap. Filling this gap and supporting greater behaviour change requires considered intervention design and targeting of messages.

Participants reported that they were less willing to change the discretionary foods and beverages they consume the most. In determining the targets of public health interventions and campaigns, there has been a tendency to target aspects of the diet that are most in need of change at a population or individual level. However, in doing so, we may be targeting foods and beverages that people are least willing to reduce. It may be worthwhile considering what people are most willing to change to set them up for success in the first instance. Although goal setting theory suggests more challenging goals tend to lead to more success, goals should also be attainable in order to promote success (28, 29). This is important as successful practice of a behaviour, and in particular early success, can enhance self-efficacy and lead to greater long-term change (30, 31). Our findings suggest that perhaps the highest ranked discretionary food or beverage category by intake may not be the best place to start, but rather starting with other categories of intake that may be less habitual and perhaps more amenable to change. Habitual behaviours or those performed regularly (such as daily or weekly) and within a relatively stable context, can be difficult to change (32). Our participants preference for changing less habitual food and beverage intake gives further support to the focus on habit strength as a target for intervention (33).

This sample of Australian adults over-consuming discretionary foods and beverages was more willing to change their intake of takeaway foods and savoury pastries, and less willing to change their intake of alcohol. This is of concern, as alcohol is by far one of the largest contributors to discretionary food and beverage intake in Australia. Almost 40% of our sample consumed more serves of alcohol per day than any other discretionary food or beverage category. Analyses of the 2011–12 National Nutrition and Physical Activity Survey found that wine and beer were in the top four discretionary foods and beverages according to energy contribution (34). Alcohol consumption is a socially and culturally ingrained behaviour in Australia, with the 2019 National Drug Strategy Household Survey showing that four in five Australian adults consumed alcohol in the previous 12 months (35). As alcohol itself contains 7 kcal of energy per gram, and most alcoholic drinks also contain energy from sugars and non-fermented starches, they are considered ‘discretionary’ in the ADG’s (1). However, as alcohol is also a social stimulant and an addictive drug, alcohol intake can be a difficult behaviour to change. Despite the lower willingness to change intake of alcohol, many people reported to be actively attempting to reduce their intake of alcohol, with 9% reporting feeling successful in doing so.

Our findings suggest that takeaway foods may be an important, and acceptable, aspect of our intake to address. Takeaway was not as commonly the highest ranked food by consumption among our sample, although it was still one of the more consumed foods among the 11 categories assessed. Takeaway, fast foods and meat pies may be more likely to be consumed as meal substitutes and therefore seen as easier to change, as there are many healthier alternatives. The preference for ‘healthy swaps’ for takeaway foods therefore makes practical sense. Takeaway food has also been typically held as an example of junk or discretionary food, meaning it may be more socially acceptable to target. Takeaway food consumption has been shown to contribute to socioeconomic inequalities in health (36, 37), making it a particularly important target for intervention. Furthermore, takeaway foods may have been overlooked as intervention targets, while the attention has been strongly on SSB’s and added sugars in recent years (14).

In terms of strategies that Australians are willing to try to reduce their intake of discretionary food and beverages, ‘days off’ was the most popular of the five strategies investigated, followed by ‘healthy swaps’. Whereas ‘elimination’ was the least preferred strategy. ‘Days off’ was particularly popular with respect to alcohol and sweet discretionary foods, such as confectionary and ice-cream. This may be in part due to the familiarity of this message as it relates to ‘alcohol-free days’, which has been promoted as a strategy for reducing alcohol intake by government (38) and non-government organisations (39). It may also relate to how and when these foods are consumed, perhaps being in addition to, rather than instead of, one’s usual main meals or snacks. Strategies involving moderation (having less discretionary foods and beverages by reducing the quantity or frequency with which they are eaten) and substitution (swapping of discretionary foods and beverages for healthy alternatives) have both been shown to be effective in reducing the overall energy contribution of discretionary food and beverages, but substitution may confer additional benefits to fibre, protein and micronutrient intakes (40). This is particularly important where discretionary foods are consumed instead of, or as part of, a nutritionally important meal such as lunch or the evening meal. Our findings show that when it comes to moderating intake, perhaps strategies targeting frequency of intake may be preferred over those targeting portion size.

In this large-scale survey, participants closely resembled the broader population in terms of weight status. Of the total sample, 33.5% were classified as having overweight and 29.5% obesity, compared with 36 and 31% as reported in the 2018 National Health Survey (6). The survey also engaged a large proportion of over-consumers of discretionary food and beverages, who were more likely to be male, to live with overweight or obesity, and not have a university education. Females and university educated adults were over-represented, which is often the case in nutrition research. The main limitation was in the measures of stage of change, willingness and dietary strategies which, while based on constructs of importance in the literature, were not previously validated or united by a single theory.

## 5 Conclusion

Australian adults are mostly willing and actively trying to make a change to their discretionary food and beverage intake. However, simply targeting the foods and beverages that are consumed in the largest quantities fails to account for how willing and ready they might be to change their intake of those groups. Messages focusing on reductions in the frequency of consumption of popular and habitually consumed discretionary foods and beverages may be more acceptable to the community than messages encouraging abstinence or portion control.

## Data availability statement

The datasets presented in this article are not readily available because ethics approval does not cover the sharing of data with a third-party. Requests to access the datasets should be directed to gilly.hendrie@csiro.au.

## Ethics statement

The studies involving humans were approved by CSIRO Health and Medical Human Research Ethics Committee Low Risk Review Panel. The studies were conducted in accordance with the local legislation and institutional requirements. The participants provided their written informed consent to participate in this study.

## Author contributions

CM: Conceptualization, Data curation, Methodology, Writing – review & editing, Formal analysis, Writing – original draft. EB: Conceptualization, Methodology, Writing – review & editing. GH: Conceptualization, Methodology, Writing – review & editing, Data curation, Project administration, Supervision.
